# Temporal changes in global soil respiration since 1987

**DOI:** 10.1038/s41467-020-20616-z

**Published:** 2021-01-15

**Authors:** Jiesi Lei, Xue Guo, Yufei Zeng, Jizhong Zhou, Qun Gao, Yunfeng Yang

**Affiliations:** 1 State Key Joint Laboratory of Environment Simulation and Pollution Control, School of Environment, Tsinghua University, Beijing, 100084 China; 2 Institute for Environmental Genomics, University of Oklahoma, Norman, OK 73019 USA; 3 Department of Microbiology and Plant Biology, University of Oklahoma, Norman, OK 73019 USA; 4 Earth and Environmental Sciences, Lawrence Berkeley National Laboratory, Berkeley, CA 94270 USA

**Keywords:** Climate-change ecology, Carbon cycle, Climate change

## Abstract

As the second-largest terrestrial carbon (C) flux, soil respiration (*R*_S_) has been stimulated by climate warming. However, the magnitude and dynamics of such stimulations of soil respiration are highly uncertain at the global scale, undermining our confidence in future climate projections. Here, we present an analysis of global *R*_S_ observations from 1987–2016. *R*_S_ increased (*P* < 0.001) at a rate of 27.66 g C m^−2^ yr^−2^ (equivalent to 0.161 Pg C yr^−2^) in 1987–1999 globally but became unchanged in 2000–2016, which were related to complex temporal variations of temperature anomalies and soil C stocks. However, global heterotrophic respiration (*R*_h_) derived from microbial decomposition of soil C increased in 1987–2016 (*P* < 0.001), suggesting accumulated soil C losses. Given the warmest years on records after 2015, our modeling analysis shows a possible resuscitation of global *R*_S_ rise. This study of naturally occurring shifts in *R*_S_ over recent decades has provided invaluable insights for designing more effective policies addressing future climate challenges.

## Abstract (web summary)

Soils hold massive amounts of carbon that hangs in the balance of microbial respiration and climate warming. Here the authors analyze a global dataset starting in 1987 and find through modeling that though soil respiration change had flatlined, recently it has resumed increasing owing to global warming.

## Introduction

In Earth’s terrestrial ecosystem, soil organic carbon (SOC) is among the largest C pools, containing two or three times more C than that in the atmosphere^1,2^. As a result, the role of soil C in natural climate solutions is evident, necessitating land-based efforts to mitigate climate changes and deliver sustainable ecosystem services^3^. The ongoing trend of climate change has stimulated the heterotrophic component of soil respiration (*R*_S_), which converts soil C to carbon dioxide (CO_2_) in the atmosphere and thus amplifies global warming^4^. *R*_S_ is affected by a complex, intertwining array of biotic and abiotic factors, among which climatic factors (i.e., temperature and precipitation)^5^ and organic matter availability (i.e., SOC)^6^ are influential. Therefore, the uncertainty regarding the magnitude and temporal dynamics of such *R*_S_ stimulation at the global scale remains one of the largest unknowns for the terrestrial C cycle and climate feedbacks. With the rapid emergence of extensive *R*_S_ studies worldwide, mining global-scale data and climate controls on *R*_S_ has only recently become available^7,8^, allowing for indispensable quantification and even prediction of global C fluxes emanating from soils^9,10^.

**Fig. 1:**
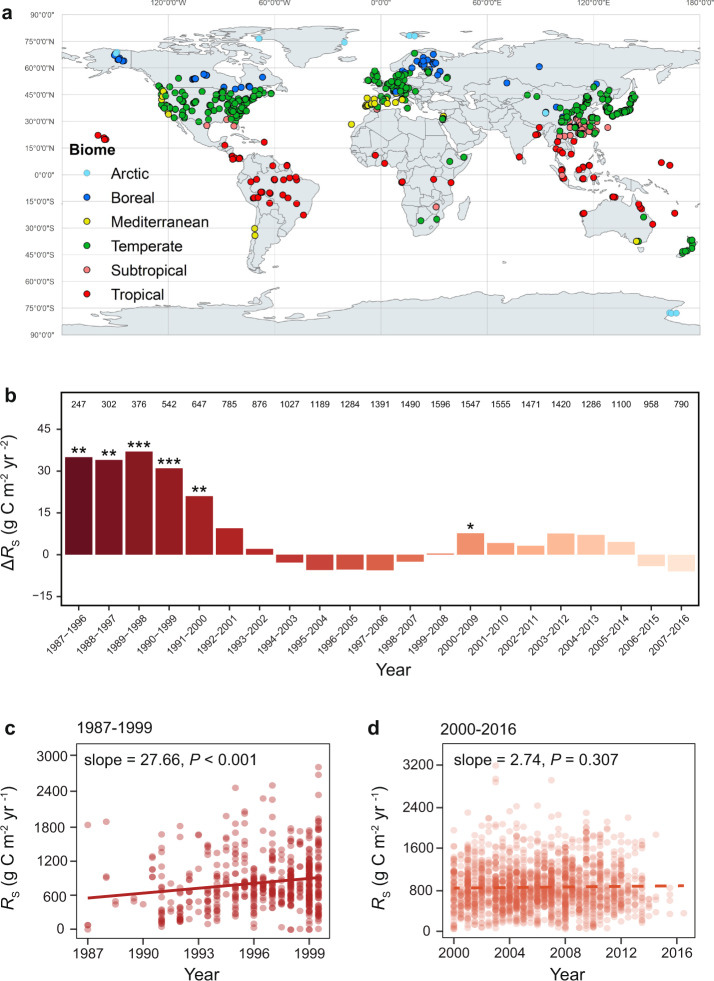
Geographical distribution of *R*_S_ data and temporal changes of *R*_S_ in different time periods. **a** A total of 2,428 observations in 693 published studies were retrieved from the SRDB, covering six types of biomes as denoted by different colors. The distribution of biomes displays clear latitude-dependent features. **b** The temporal trend of *R*_S_ changes in each decade based on the moving subset window analysis. The bars indicate the rates of *R*_S_ changes per decade, calculated as average annual *R*_S_ change (Δ*R*_S_). The number above each bar refers to the number of *R*_S_ records belonging to the subset window. The color scale of the bars corresponds to the colors representing different periods in (**c**, **d**).***P < 0.001, **P < 0.010, *P < 0.050. **c** The relationship between *R*_S_ and year in 1987–1999. **d** The relationship between *R*_S_ and year in 2000–2016. The slope of linear regression indicates the rate of *R*_S_ changes in different time periods. The dot density represents data density at each point. Solid lines indicate significant trends (*P* < 0.050), while dashed lines indicate insignificant trends (*P* ≥ 0.050).

Here, we examined the temporal changes of *R*_S_ from the version 20200220a of the global *R*_S_ database (SRDB) downloaded from github.com/bpbond/srdb^10^, which were obtained by infrared gas analyzers and gas chromatographic techniques from non-agricultural ecosystems without experimental manipulation. A total of 2,428 annual *R*_S_ data measured worldwide from 1987 to 2016 were collected from 693 studies (Fig. 1a), over half of which were not included in the SRDB used by the last major *R*_S_ study^7^. We aim to address the following questions: (i) how *R*_S_ has changed in the last three decades; and (ii) what factors best explain the temporal changes of *R*_S_. Our observational and modeling results indicate that global *R*_S_ rise has significantly slowed down in the early 21st century. Temporal *R*_S_ dynamics vary by different biomes, latitudes, and ecosystems, with *R*_*S*_ decreasing in the tropical and temperate biomes but increasing in the boreal and Arctic biomes. In contrast, global *R*_h_ has steadily increased over the time period of 1987 to 2016, suggesting that there is a high risk of soil C loss, particularly in high latitudes of the Earth.

## Results and discussion

### Temporal trends of *R*_S_ observations

Global *R*_S_ was rising during 1987–2016 (*P* = 0.048; Supplementary Fig. 1), which was consistent with the previous studies^7,10^. However, the temporal trend and magnitude may be contingent on the choice of the start and end years^11^ in our study. Therefore, we performed a moving subset window analysis (see details in “Methods”)^12^ and found that the rates of *R*_S_ changes (i.e., the slope between *R*_S_ and year) were significantly positive in the early years but remained largely unchanged in the later years (Fig. 1b). The results were unaffected by possible data anomalies, as verified by robust regression using the Theil-Sen estimator (Supplementary Fig. 2a). The results were also robust when controlling for the variability of climate conditions (i.e., mean annual temperature (MAT) and mean annual precipitation (MAP)), latitude, altitude, measurement method, ecosystem, biome type, developmental stage of the ecosystem and SOC stocks^7,10^ (*P* = 0.015 for the year quadratic effect in a linear model; Table 1).

**Table 1:** Summary of the effects in the linear model of *R*_S_ during 1987–2016^a^.

Effect	Degree of freedom	F	*P*
Year	1	9.063	0.003
Year^2^	1	5.964	0.015
Biome	5	6.444	0.001
Latitude	1	870.373	0.001
ΔMAT	1	9.005	0.003
Year × Biome	5	3.077	0.009
Year × Latitude	1	4.925	0.027
Year × SOC	1	18.469	0.001
Biome × ΔMAT	5	3.887	0.002
Biome × ΔMAP	5	2.480	0.030
Biome × ΔMAT × ΔMAP	5	6.978	0.001

^a^The statistically significant (*P* < 0.050) effects are shown. Effects tested in the linear model include the year of *R*_S_ measurement and its quadratic form (Year^2^), biome (tropical, subtropical, Mediterranean, temperate, boreal and Arctic), measurement method, latitude, altitude, stage (aggrading or mature ecosystem), ecosystem type (i.e., forest, grassland, savanna, shrubland, and wetland), SOC stock, and climatic factors (MAT, MAP, ΔMAT, and ΔMAP). The “×” sign denotes an interaction term. All terms and more detailed information are shown in Supplementary Table 2.

A closer examination showed that *R*_S_ increased at a rate of 27.66 g C m^–2^ yr^–2^ (equivalent to 0.161 Pg C yr^–2^) from 1987–1999 (*P* < 0.001; Fig. 1c), but became unchanged in 2000–2016 (*P* = 0.307; Fig. 1d), suggesting a halt of global *R*_S_ rise in the early 21st century. This finding was consistent with a top-down global estimate of reduced ecosystem respiration during a warming hiatus^13^, characterized by a slowdown of global surface warming during 1999–2014^2,11^. Arising through combined effects of internal decadal variability, uptake of heat by the oceans, negative radiative forcing from anthropogenic sulfate aerosol emissions, and solar activity; the warming hiatus is characterized by a slowdown rather than a complete halt in global temperature rises^11^. Therefore, the halt of global *R*_S_ rise during the warming hiatus implies that warming rate is unlikely to be the sole determinant of *R*_S_ changes.

**Fig. 2:**
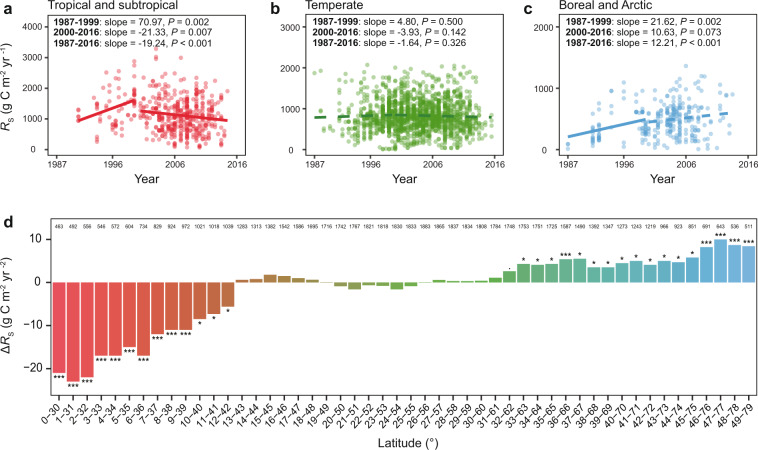
Dependence of *R*_S_ changes on biomes and latitudes. **a**–**c** The relationships between *R*_S_ and the year in 1987–1999 and 2000–2016 in tropical and subtropical biomes (**a**), temperate and Mediterranean biomes (**b**), and boreal and Arctic biomes (**c**). The slope of linear regression indicates the rate of *R*_S_ changes in different biomes. The dot density represents data density at each point. Solid lines indicate significant trends (*P* < 0.050), while dashed lines indicate insignificant trends (*P* ≥ 0.050). **d** Latitude dependence of *R*_S_ changes based on the moving subset window analysis. Each window includes a subset of *R*_S_ data within a 30° latitude interval and moves forward by 1° step. The bars represent the rates of *R*_S_ changes in different latitudes, calculated as average annual *R*s change (Δ*R*s) in 1987–2016. The number above each bar refers to the number of *R*_S_ records belonging to the subset window. The color scale of the bars corresponds to the colors representing different biomes in (**a**–**c**). ***P < 0.001, **P < 0.010, *P < 0.050.

The current *R*_S_ data obtained in the SRDB cover most of the geographic regions of the world (from 78.02° S to 78.17° N) and biome types (tropical, subtropical, temperate, Mediterranean, boreal, and Arctic biomes). Previous empirical experiments in both field and laboratories indicated that temporal changes of *R*_S_ varied by biomes^14–16^. Similarly, we found a significant year × biome interaction (*P* = 0.009 in a linear model; Table 1). During 1987–1999, *R*_S_ increased at a staggering rate of 70.97 g C m^–2^ yr^–2^ (*P* = 0.002) in tropical and subtropical biomes, remained unchanged (*P* = 0.500) in temperate biomes, while it increased at a rate of 21.62 g C m^–2^ yr^–2^ (*P* = 0.002) in boreal and Arctic biomes (Fig. 2a–c). During 2000–2016, *R*_S_ was shifted to a decreasing rate of −21.33 g C m^–2^ yr^–2^ (*P* = 0.007) in tropical and subtropical biomes, but remained unchanged (*P* > 0.050) in temperate, boreal, and Arctic biomes. As biomes and climate conditions are latitude-dependent (Supplementary Fig. 3)^17^, the rates of *R*_S_ changes were also latitude-dependent (*P* = 0.027 for the year × latitude interaction; Table 1), being negative in lower latitudes but positive in higher latitudes (Fig. 2d). This finding was robust to outliers, as verified by robust regression (Supplementary Fig. 2b).

The *R*_S_ data in SRDB are mainly collected from forests and grasslands, which cover 70% of the land surface^18,19^. *R*_S_ in grasslands worldwide decreased at a rate of 13.75 C m^–2^ yr^–2^ during 1987–2016 (*P* = 0.011, Supplementary Table 1), possibly owing to limited SOC and dry climate conditions typical in most grasslands^20^. In contrast, *R*_S_ in forests worldwide remained unchanged (*P* > 0.050). When forests were divided into evergreen forests, deciduous forests, and mixed forests, we found that *R*_S_ remained unchanged in 1987–1999 in all three forest types (*P* > 0.050, Supplementary Table 1). In 2000–2016, *R*_S_ increased (*P* < 0.001) at a rate of 18.08 C m^–2^ yr^–2^ in evergreen forests, remained unchanged in deciduous forests, but decreased (*P* = 0.040) at a rate of 20.13 C m^–2^ yr^–2^ in mixed forests. Those results supported that the temporal changes of *R*_S_ were negative in the low and middle latitudes, wherein grasslands and mixed forests are abundant natural ecosystems^21^.

### Influence of climatic factors and SOC on temporal changes of *R*_S_

**Fig. 3:**
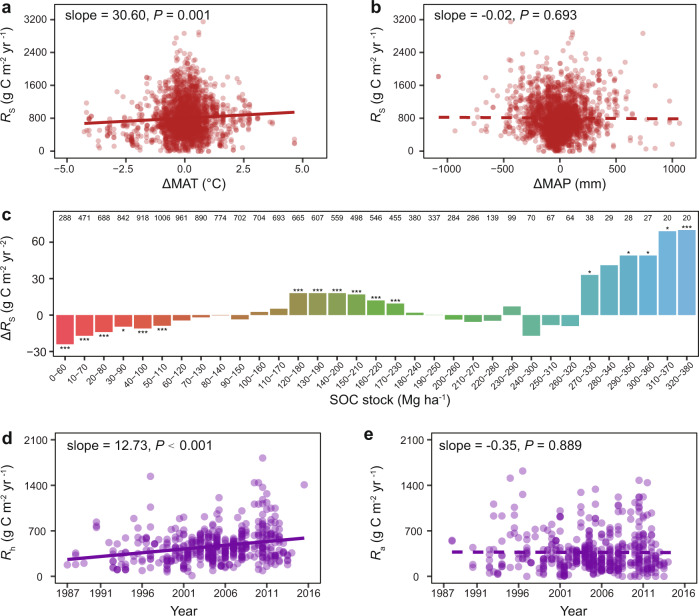
Covariates of *R*_S_ changes. **a**, **b** The relationship between *R*_S_ and temperature anomaly (ΔMAT) (**a**) and precipitation anomaly (ΔMAP) (**b**). **c** The moving subset window analysis of *R*_S_ changes in different SOC stock levels. Each window includes a subset of *R*_S_ data whose SOC stocks are within a range of 60 Mg ha^–1^ and moves forward by 10 Mg ha^–1^ step. The bars represent the rates of *R*_S_ changes in different ranges of SOC stocks calculated as average annual *R*_S_ change (Δ*R*_S_) in 1987–2016. The number above each bar refers to the number of *R*_S_ records belonging to the subset window. ***P < 0.001, **P < 0.010, *P < 0.050. **d**, **e** Temporal trends of global *R*_h_ (**d**) and *R*_a_ (**e**) in 1987–2016. The slope of linear regression indicates the rate of changes of *R*_h_ or *R*_a_. The dot density represents data density at each point. Solid lines indicate significant trends (*P* < 0.050), while dashed lines indicate insignificant trends (*P* ≥ 0.050).

Potential, non-mutually-exclusive mechanisms causing the slowdown of global *R*_S_ rise include shifts in the complex interactions between climate factors^2^, SOC availability^22^, and different sensitivities of plant and microbial respiration due to climate change^23^. To address the influence of climate factors, we calculated temperature and precipitation anomalies (ΔMAT and ΔMAP, the yearly deviations of those variables from their mean values in 1987–2016)^7^ for each *R*_S_. Consistent with the trend of global warming^24,25^, ΔMAT, a mean of temperature anomalies of the SRDB sites, showed an overall temporal trend of 0.18 °C increase per decade (*P* < 0.001; Supplementary Fig. 4a) from 1987–2016. The rising rate of ΔMAT was 0.21 °C per decade in the early period (1987–1999) but dropped to 0.14 °C per decade in the later period (2000–2016). ΔMAT decreased in lower latitudes (Supplementary Fig. 4c) but was similar between 1987–1999 and 2000–2016 in higher latitudes (Supplementary Fig. 4d), as verified by both satellite-based tropospheric data and global surface temperature data^26^. ΔMAT was positively correlated with *R*_S_ after controlling for other effects (*P* = 0.003; Table 1 and Fig. 3a), consistent with the finding of the SRDB prior to 2008^7^, and suggested that ΔMAT had good explanatory power for the global *R*_S_ rise. In contrast, there was no significant correlation between ΔMAP and *R*_S_ (*P* = 0.430; Fig. 3b and Supplementary Table 2), though a significant temporal trend of ΔMAP was observed at the global scale (*P* < 0.001; Supplementary Fig. 4b). It is probable that the slowdown of global warming stimulates activities of soil decomposers (i.e., bacteria, fungi, protists, and metazoan) and plant root respiration^27^ to a lesser extent, which in turn slowed soil C decomposition and *R*_S_. Also, plant growth can be affected by the slowdown of global warming^13^, reducing plant root exudation that produces fresh soil C inputs and consequently constraining the increase of global *R*_S_ by lower priming effects.

We observed a significant two-way interaction involving ΔMAT and biome (*P* = 0.002 for the biome × ΔMAT interaction; Table 1 and Supplementary Fig. 5a–c), indicating that the effect of ΔMAT on *R*_S_ varied by biomes. Similarly, ΔMAP was only positively correlated with *R*_S_ in boreal biomes, but not in tropical and temperate biomes (Supplementary Fig. 5d–f). As warming gives rise to less precipitation in temperate and tropical regions^2^, the positive response of *R*_S_ to warming can be constrained by soil moisture. Warming can also reduce microbial carbon-use efficiency^28^. Although warming is generally believed to increase *R*_S_^28^, low growth efficiencies of microbes at higher temperatures in tropical and temperate regions could decrease *R*_S_ by reducing microbial capacity to decompose organic resources^29^. In contrast, *R*_S_ can be stimulated by warming in boreal and Arctic ecosystems due to richer soil C stocks, wetter environments, and greater catabolic rates of microbes^24,25^. Labile C-rich woody shrubs and moss are the dominant plant species in boreal and Arctic systems. The fresh C input could accelerate the decomposition of more recalcitrant forms of SOC through the biological priming mechanisms^30^. Indeed, high temperature sensitivity of *R*_S_ in cold biomes has already been known for nearly half a century^31^.

The rates of *R*_S_ changes are related to soil C stocks^22^. Despite this ongoing dispute, previous warming experiments showed that warming-induced soil C loss might be related to the standing soil C stock, with more C losses occurring in soils with higher C stocks^32^. Consistent with this, we found that the rates of *R*_S_ changes were correlated to SOC (Fig. 3c), which was robust after controlling for the variability of climate conditions (MAT and MAP), latitude, altitude, measurement method, ecosystem, biome type, and developmental stage of the ecosystem (*P* < 0.001 for the year × SOC interaction in a linear model; Table 1). The rates of *R*_S_ changes were negative in the SOC range of 0–100 Mg ha^–1^ (Slope = −10.39 g C m^–2^ yr^–2^, *P* < 0.001; Supplementary Fig. 6a) but became positive in the SOC range of 100–180 Mg ha^–1^ (Slope = 9.53 g C m^–2^ yr^–2^, *P* < 0.001; Supplementary Fig. 6b). This finding explains the ecosystem dependence of *R*_S_ changes due to the rates of *R*_S_ changes being negative in mixed forests and grasslands typically of small SOC stocks^33,34^, but became positive in SOC-rich soils where evergreen forests are mainly present^35^. Notably, experimental warming of temperate forest soils gave rise to a nonlinear, four-phase *R*_S_ pattern related to periods of compositional and physiological changes in the microbial community^36^. As a result, there was a shift toward the decay of more recalcitrant C substrates upon depleting microbial accessible C pools^36,37^. This observation provides an important mechanism for temporal changes of *R*_S_ (Fig. 1), necessitating the need for deeper soil C component analysis in the future.

Unexpectedly, the *R*_S_ changes were insignificant in the SOC range of 180–270 Mg ha^–1^ (Slope = −0.73 g C m^–2^ yr^–2^, *P* = 0.807; Supplementary Fig. 6c), in which 77.2% of *R*_S_ data were observed in temperate biomes. Chemical mineralization rates are high in temperate regions, protecting SOC stocks from microbial access and thus reducing soil C decomposition^22^. Additionally, saturated soil moisture might create anaerobic microenvironment in some humid temperate regions, which potentially inhibits aerobic respiration. The rates of *R*_S_ changes were the highest when the SOC was above 270 Mg ha^–1^ (Slope = 58.22 g C m^–2^ yr^–2^, *P* < 0.001; Supplementary Fig. 6d), in which 61.8% of the *R*_S_ data were observed in boreal or high-latitude regions. Recent decades have witnessed the most remarkable warming and permafrost thaw in those C-rich regions, which releases previously frozen organic C to be accessible for microbial decomposition^38^.

**Fig. 4:**
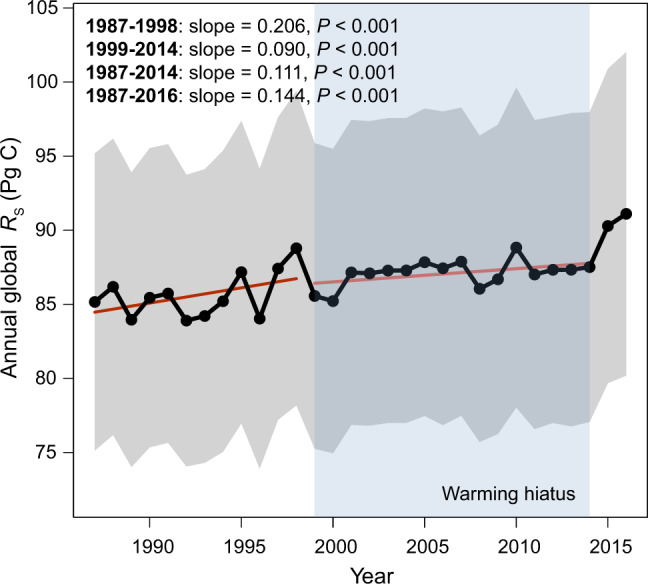
Estimated annual global *R*_S_. Slopes and significances of linear regressions between annual global *R*_S_ from 1000 Monte Carlo trials and year are shown. Regression lines of *R*_S_ for the year in 1987–1998 and 1999–2014 are shown in red. The gray region denotes the standard deviations of annual global *R*_S_ from Monte Carlo simulations. The filled blue area denotes the hiatus period of global warming (i.e., 1999–2014).

It is important to examine the integrative effect of climatic factors on global *R*_S_ dynamics through modeling. To estimate annual global *R*_S_ during 1987–2016, we adopted a Monte Carlo approach based on a fitted multivariate model with gridded time-series climate data. The estimated mean value of annual global *R*_S_ was 85.6 Pg C in 1987–1998 (Fig. 4), which was increased to 87.5 Pg C in 1999–2016, suggesting a globally rising annual *R*_S_. Our estimated global *R*_S_ values were close to those derived by other models^7,39,40^, showing consistency across different models. Annual global *R*_S_ increased at a rate of 0.21 Pg C yr^–1^ in 1987–1998 (t_11,998_ = 7.604, *P* < 0.001; Fig. 4), but slowed down to the rate of 0.09 Pg C yr^–1^ (t_15,998_ = 5.008, *P* < 0.001) in 1999–2014. Our model predicted a sharp increase in annual global *R*_S_ after 2014 (Fig. 4), suggesting that those warmest years on records could strongly stimulate annual global *R*_S_. However, our prediction can only be tested when we have enough post-2014 observational data in the future.

### Temporal trends of heterotrophic and autotrophic respiration

*R*_S_ is comprised of heterotrophic respiration (*R*_h_) of microbes and autotrophic respiration (*R*_a_) of plant roots and associated rhizosphere microbes, which are also documented in the SRDB (468 observations for *R*_h_ and 473 observations for *R*_a_, though they are relatively limited in sample size and subject to larger errors owing to difficulties to measure *R*_h_ and *R*_a_^7^). Therefore, we could use them to explore the possibility of soil C loss. Consistent with observations in global *R*_S_ (Supplementary Fig. 1), global *R*_h_ showed a positive temporal trend during 1987–2016 (*P* < 0.001; Fig. 3d), whereas *R*_a_ did not change over time (*P* = 0.889; Fig. 3e) after controlling for climate conditions (MAT and MAP), biome, latitude, altitude, measurement method, partitioning method, ecosystem, developmental stage of the ecosystem, and SOC stocks (Supplementary Table 3). Similar to observations in *R*_S_ (Table 1), *R*_a_ and *R*_h_ were also significantly correlated with biome, latitude, altitude, measurement method, and partitioning method (Supplementary Table 3). Those results supported the previous observation of increasing *R*_h_: *R*_S_ ratios in recent decades^10^ and were consistent with a meta-analysis showing that *R*_a_, but not *R*_h_, had thermal acclimation to long-term warming^41^. It is unlikely for *R*_h_ to fully acclimate to warming since depletion of labile C pools in soils will irreversibly change microbial community composition, shift microbial carbon use efficiency, and reduce microbial biomass^36^. Therefore, the slowdown of global *R*_S_ rise might be accompanied by soil C loss mediated by *R*_h_, and thus amplifies the positive feedback between soil C and atmosphere.

### Limitations and outlook

It is important to note several limitations of this study. First, the SRDB is a collection of published studies of in-situ soil respiration. Consequently, most measurements are from mid-latitudes of the Northern Hemisphere (61.7%), and measurements in forests accounted for 77.5% of the total sample size^7,10^. The relatively fewer data from low- and high-latitudes suggested a need for more research to investigate these regions in the future. Hot dry and cold dry biomes (e.g., Central Australia, African Sahara, the Middle East, and Russia) are underrepresented in the study, owing to a lack of extensive research. Global warming is projected to accelerate drying in the tropics but increase precipitation and atmospheric humidity in high-latitudes^42,43^, so we suspect that the inclusion of more data from low- and high-latitude arid regions in the future can affect our major findings but is unlikely to refute them. Additionally, there is a paucity of observational data after 2014. It is thus unclear whether the slowdown of global *R*_S_ rise persists in more recent years. Since SRDB is continuously updated (a new version of SRDB is now available^44^), it is expected that our prediction could be verified with increasing amount of data covering more recent periods. Second, some confounding factors (i.e., soil pH, moisture, and vegetation) are not accounted for in this study, which could affect our results. Third, similar to any observational analysis, the underlying bias caused by the spatial and temporal inconsistency of *R*_S_ data is intractable for causality inference, which can be addressed by manipulative experiments^32,36^. Finally, it is noteworthy that the scope of this study is restricted to terrestrial ecosystems, a more holistic picture of global C cycling could be provided by incorporating changes in other major C fluxes from principal C sinks such as Oceans.

In a nutshell, we showed that global *R*_S_ increased in 1987–1999 but became largely unchanged in 2000–2016, leveraging a rapidly expanding database comprised of global in situ *R*_S_ measurements in natural ecosystems. Our analysis of large-scale terrestrial respiration data allows us to see past the conflicting results from single-site studies by capturing global patterns in a warmer world. The slowdown of global *R*_S_ rise is very likely to be resuscitated since global-mean surface air temperature has set new records again since 2015. However, we predict that global *R*_S_, under the joint influence of temperature anomalies and soil C stock, would not rebound rapidly, offering a testbed for hindcasting when newer data are included in the SRDB. Our analysis directly addresses the long-held concern about the positive land C-climate feedback that could accelerate planetary warming in the 21st century, which is critical for ecological forecasting and climate policy-making. Given the huge impacts of warming on large soil C storage in cold regions^13–15^, the stronger increase of *R*_S_ in high latitudes warrants more efforts focusing on climate change research in these regions.

## Methods

### Global *R*_S_ dataset

The Global Soil Respiration Database (SRDB) consists of seasonal and annual *R*_S_, *R*_h_, and *R*_a_ records from more than 10,000 published studies to date, which we filtered according to the criteria below. The version of SRDB has been updated over time, of which detailed information has been described in the previous studies^7,45^. Here, we retrieved *R*_S_, *R*_h_, and *R*_a_ from the version 20200220a of the SRDB downloaded from github.com/bpbond/srdb. To ensure data consistency and accuracy, we used only the respiration records that (1) reported annual measurements; (2) had basic spatial and temporal information (longitude, latitude, and measurement years); (3) were measured from non-agricultural ecosystems without experimental treatments (i.e., nitrogen addition, warming, precipitation alternation); and, (4) only used infrared gas analyzers or gas chromatography for CO_2_ fluxes measurements, given that other measurement methods, such as Alkali absorption and soda-lime measurements, could potentially misestimate soil respiration. Because the standard method of *R*_S_ measurements, i.e., the use of infrared gas analyzers or gas chromatography, was not widely used before 1987, only a few *R*_S_ records were collected in 1961–1986. Thus, we only used *R*_S_ records after 1986 in this study. To minimize the influence of “extreme” values, we identified outliers as the measurements of *R*_S_ exceeding −3 or +3 standard deviations from the population mean. Consequently, 41 *R*_S_ data (1.8% of total data) were removed from the dataset.

A total of 2,428 *R*_S_ data in 1987–2016 were obtained from 693 studies, which spanned across a large latitudinal gradient (78.02° S-78.17° N) and covered most ecosystem types, including forest, grassland, shrubland, wetland, and desert. Over half of those data were included in the SRDB after the last major *R*_S_ study of SRDB^45^. The geographic locations of these *R*_S_ data are visualized using the mapping tools in ArcGIS 10.2 (ESRI 2013; Environmental Systems Research Institute, Redlands, CA, USA)^46^, with a global map downloaded from https://www.naturalearthdata.com/downloads/110m-cultural-vectors/ as the base map. Additionally, 468 *R*_h_ records from 158 studies and 473 *R*_a_ records from 157 studies in 1987–2016 were retrieved from the SRDB under the criteria as *R*_S_.

### Environmental parameters

Soil respiration in the SRDB is well-matched with many parameters, including climate types, ecosystem types, geography, spatial (longitude and latitude) and temporal (measurement years and duration of the study) information, experimental design, measurement methods for CO_2_ fluxes, as well as methods used to partition *R*_S_ source fluxes into *R*_h_ and *R*_a_^45^. In a few cases, some parameters, such as spatial or temporal information, are missing from the SRDB for certain *R*_S_ records. Therefore, we collected the missing values from the corresponding studies or other databases^47^.

The climatic datasets for terrestrial air temperature and precipitation were downloaded from the Center for Climatic Research at the University of Delaware (climate.geog.udel.edu/~climate/html_pages/ download.html). We used the most recently updated Gridded Monthly Time Series database (version 5.01), which holds a 0.5-degree latitude × 0.5-degree longitude global grid of air temperature and precipitation in both monthly and annual time series from 1900/01–2017/12^48^. Mean annual temperature (MAT) and mean annual precipitation (MAP) were calculated and matched with *R*_S_ records based on their latitude and longitude coordinates and temporal information. Some studies have reported *R*_S_ records measured through more than one year or average annual *R*_S_ across multiple years, so we calculated average temperature and precipitation within the measurement period by monthly data from climatic datasets and used them as MAT and MAP in this study. For instance, MAT for a 1.5-year record referred to the 18-month average temperature of the experimental site. Likewise, other parameters were also derived and spatiotemporally matched with *R*_S_ data.

To quantify temperature and precipitation anomalies based on climate records of the past decades^7,49,50^, ΔMAT and ΔMAP were defined by the following equations:

1 $$\Delta {\mathbf{MAT}} = {\mathbf{MAT}} - \overline {{\mathbf{MAT}}}$$

2 $$\Delta {\mathbf{MAP}} = {\mathbf{MAP}} - \overline {{\mathbf{MAP}}}$$

where MAT and MAP are the annual temperature and precipitation at a particular site and at a certain time, respectively; and $\overline {{\mathrm{MAT}}}$ and $\overline {{\mathrm{MAP}}}$ are the mean of annual temperature and precipitation at the same site across 1987–2016, respectively.

Only a few datasets are available for assessing the spatial-temporal distribution of SOC stock^51,52^. Here, we generated a global SOC dataset by using the SoilGrids 250 m dataset (soilgrids.org)^53^, which offers a collection of global standard numeric soil property at a spatial resolution of 250 m. ArcGIS 10.2 was used to extract topsoil (0–30 cm) SOC stock according to the longitude and latitude coordinates of *R*_S_. Since 4% of the *R*_S_ observations lacked SOC data in the SoilGrids dataset, we substituted each missing value by the median of SOC stock data of the same ecosystems. We also obtained the altitude for each location of *R*_S_ data from the GPS Visualizer’s Elevation Lookup Utility^54^.

### Statistical analyses

All statistical analyses were carried out in R version 3.6.1^55^ with the package “stats” unless otherwise indicated. A linear model weighted by years of *R*_S_ measurement^45^ was used to investigate the effects of year and its quadratic form (i.e., Year^2^)^56^, climatic factors (MAT, MAP, ΔMAT, and ΔMAP), geographic factors (latitude and altitude) and other factors (biome, measurement method, ecosystem, developmental stage of the ecosystem, and SOC stock) on *R*_S_:

3 $${\boldsymbol{R}}_{\mathbf{S}} \sim 	\,{\mathbf{Year}}^{\mathrm{2}} + {\mathbf{Year}} \times {\mathbf{Method}} + {\mathbf{Year}} \times {\mathbf{Latitude}}\\ 	+\, {\mathbf{Year}} \times {\mathbf{Altitude}} + {\mathbf{Year}} \times {\mathbf{Stage}}\\ 	+\, {\mathbf{Year}} \times {\mathbf{Ecosystem}} + {\mathbf{Year}} \times {\mathbf{SOC}} + {\mathbf{Year}} \times {\mathbf{Biome}}\\ 	+\, {\mathbf{MAT}} \times {\mathbf{MAP}} \times {\mathbf{Biome}} + \Delta {\mathbf{MAT}} \times \Delta {\mathbf{MAP}} \times {\mathbf{Biome}}$$

where *R*_S_ is annual soil respiration, Year is the year for *R*_S_ measurement, Method refers to the method of CO_2_ flux quantification (infrared gas analyzers or gas chromatography), Latitude and Altitude are the geographic locations of observation sites, Stage refers to the developmental stage of the ecosystem (i.e., aggrading or mature), Ecosystem refers to ecosystem types, including forest, grassland, savanna, shrubland, wetland and others, SOC is the topsoil (0–30 cm) SOC stock at the observation sites, Biome includes tropical (*n* = 250), subtropical (*n* = 244), temperate (*n* = 1551), Mediterranean (*n* = 93), boreal (*n* = 269) and Arctic (*n* = 21) biomes, MAT, MAP, ΔMAT, and ΔMAP are annual temperature, precipitation and their anomalies as defined above, and × indicates a term interaction. For parallel linear model analyses of *R*_h_ and *R*_a_, we added the term “Year × Partitioning method” into the formulas, where partitioning method refers to the method used to partition *R*_h_ from *R*s (i.e., exclusion, comparison, isotope, to name a few). Analysis of variance (ANOVA) was conducted after the linear model analyses to generate type I sum of squares, F statistics, and *P* values for each term.

We adopted the method of exhaustion to identify the breakpoint from multiple choices of years. Owing to the scarcity of measurement data in earlier years that can cause data anomalies, the year 1996 was set as the first possible breakpoint. Each year from 1996 to 2015 was tested as the potential breakpoint. We used different homogeneity tests (Buishand Range Test, Buishand *U* Test, and Standard Normal Homogeneity Test) to identify the change point based on *R*s change rates in the first period corresponding to different breakpoint years, which consistently showed a breakpoint between 1999 and 2000 (Supplementary Tables 4 and 5). Accordingly, we further divided all *R*s data into two non-overlapping time periods: the early period (1987–1999, *n* = 553), and the later period (2000–2016, *n* = 1,875). The linear regression analysis was used to examine the relationships between *R*_S_ and year in both time periods, between *R*_S_ and year across three groups of biomes: Tropical and Subtropical (*n* = 494), Temperate and Mediterranean (*n* = 1644) and Boreal and Arctic (*n* = 290), between *R*_S_ and climatic parameters (ΔMAT and ΔMAP). The slopes of the regressions represented the magnitude of *R*_S_ changes in response to variables of interest (i.e., year, temperature, and precipitation changes). Also, we examined the temporal trends of *R*_S_, *R*_h,_ and *R*_a_ in 1987–2016 using the same linear regression analysis.

To generate robust, reliable estimates of the temporal trend of *R*_S_ in 1987–2016, we conducted a one-dimensional moving subset window analysis^12,57^. Essentially, all *R*_S_ data were arranged in ascending order of the year of *R*_S_ measurement. The resulting datasets were iteratively divided into subsets, each comprising ten years of *R*_S_. Consequently, the first subset contains the first ten years of *R*_S,_ and the last subset contains the last ten years of *R*_S_. After the first subset was identified, the second, third, and subsequent subsets were formed by dropping the data from the earliest year in the previous subset and adding one-year *R*_S_ data following the end year of the previous one. Then, the linear regression between *R*_S_ and year was performed within each subset. The slope and *P*-value of the linear regression were the rate of *R*_S_ changes and its significance in the corresponding ten years, respectively. In the final diagram, the rate of *R*_S_ changes per decade and its significance were plotted against the subset order. To ensure that the trends in moving window analyses were not affected by anomalous data, we calculated the change rate of *R*s within each window using the Theil-Sen estimator with R package “mblm.” Theil-Sen estimators were also calculated for all regressions of subsequent moving window analyses.

The moving subset window analysis was used to examine the rates of *R*_S_ changes in the latitudinal subsets or SOC stock ranges. All *R*_S_ data were rearranged in ascending order of latitude. The resulting datasets were iteratively divided into latitudinal subsets, each comprising 30° with the corresponding *R*_S_ and 1° as the spacing between adjacent latitudinal subsets. The linear regression analysis between *R*_S_ and year was performed for each latitudinal subset. In the moving window analysis of SOC stock, all *R*_S_ data were rearranged in ascending order of SOC stock. The resulting datasets were iteratively divided into subsets, each comprising 60 Mg ha^–1^ with the corresponding *R*_S_ and 10 Mg ha^–1^ as the spacing between adjacent subsets. In each SOC stock subset, we performed a linear regression between *R*_S_ and year to calculate the slope and the significance. A few subsets of *R*_S_ data whose corresponding SOC stocks were over 380 Mg ha^–1^ had very small data sizes (less than 10 *R*_S_ data). Consequently, we set 380 Mg ha^–1^ as the upper limit of SOC moving subset windows. Finally, a total of 50 subsets were reported for latitudinal windows, and 33 subsets were reported for SOC stock windows.

We adopted a Monte Carlo approach for estimating annual global *R*_S_, following Bond-Lamberty et al.^7^. To this end, we initialized a linear model using climatic factors as inputs to fit SRDB observations:

4 $$\sqrt {{\boldsymbol{R}}_{\mathbf{S}}} \sim 	\,{\mathbf{MAT}} + {\mathbf{MAT}}^2 + {\mathbf{MAP}} + {\mathbf{MAP}}^2\\ 	\,+\, {\mathbf{MAT}} \times {\mathbf{MAP}} + {\mathbf{MAT}} \times \Delta {\mathbf{MAT}} + {\mathbf{MAP}} \times \Delta {\mathbf{MAP}}$$

A stepwise regression based on the Akaike information criterion was used to select a simplified model formula because adding more variables may introduce larger uncertainties of predictions. We then used a Monte Carlo method (*N* = 1,000) to estimate annual global *R*_S_. For each Monte Carlo trial, new model parameters were randomly sampled from the probability distribution of each model parameter characterized by a mean value and standard deviation generated by the selected simplified model. Using gridded time-series data of temperature, precipitation, and their anomalies as input variables, grid-cell annual *R*_S_ values were generated from 1,000 random modelings for each year of 1987–2016. Annual global *R*_S_ was calculated by the sum of the product of each *R*_S_ value and the corresponding land area for each grid cell. The areas of grid cells were calculated based on the latitude at the upper boundary of the cell. Only the *R*_S_ values from grid cells covering terrestrial ecosystems were considered to be meaningful. Means and standard deviations of annual global *R*_S_ were then computed based on values generated by random models. We used linear regressions to analyze the temporal trends of annual global *R*_S_ during 1987–1998, 1999–2014, 1987–2014, and 1987–2016.

## Supplementary information

Supplementary Information

## Data Availability

All data in this study from the global *R*_S_ database (SRDB) is publicly accessible at https://github.com/bpbond/srdb. Time series data of terrestrial temperature and precipitation datasets are available at http://climate.geog.udel.edu/~climate/html_pages/download.html. Global SOC stock data are available at https://data.isric.org/geonetwork/srv/eng/catalog.search#/metadata/ea80098c-bb18-44d8-84dc-a8a1fbadc061.

## Code availability

The R code for statistical analysis, licensed under the “GNU Affero General Public License” version 3, and datasets in support of these findings are available at https://github.com/LeiJiesi/Global_Soil_Respiration_Analysis. The code is comprised of the following main steps: (1) screens appropriate *R*_S_, *R*_h_ and *R*_a_ records from SRDB database and matches with environmental parameters, (2) fits multivariate models to soil respiration data, (3) conducts moving window analyses along temporal, latitudinal, and SOC stock gradients, (4) performs linear regressions between soil respiration and co-variates using OLS and Theil-Sen estimators, (5) predicts global annual *R*_S_ changes with Monte Carlo simulations.
