# Author Correction: Temporal changes in global soil respiration since 1987

**DOI:** 10.1038/s41467-021-22014-5

**Published:** 2021-03-16

**Authors:** Jiesi Lei, Xue Guo, Yufei Zeng, Jizhong Zhou, Qun Gao, Yunfeng Yang

**Affiliations:** 1 State Key Joint Laboratory of Environment Simulation and Pollution Control, School of Environment, Tsinghua University, Beijing, 100084 China; 2 Institute for Environmental Genomics, University of Oklahoma, Norman, OK 73019 USA; 3 Department of Microbiology and Plant Biology, University of Oklahoma, Norman, OK 73019 USA; 4 Earth and Environmental Sciences, Lawrence Berkeley National Laboratory, Berkeley, CA 94270 USA

**Keywords:** Climate-change ecology, Carbon cycle, Climate change

Correction to: *Nature Communications* 10.1038/s41467-020-20616-z, published online 15 January 2021.

The original version of this Article contained errors in Figure 1 and Figure 3 axes labels. In the y-axis of Figure 1d, *ΔR*_s_ should read *R*_s_*;* in the y-axis of Figure 3d *R*_s_ should read *R*_h_; in the y-axis of Figure 3e *R*_s_ should read *R*_a_. This has been corrected in both the PDF and HTML versions of the Article.

