# Supplementary material for: Temporal changes in global soil respiration since 1987
Source: Nat Commun. 2021 Jan 15;12:403. doi: 10.1038/s41467-020-20616-z (PMC7810831; doi:10.1038/s41467-020-20616-z)
Supplement: Supplementary file 1 — Supplementary Information [file 41467_2020_20616_MOESM1_ESM.pdf]

Supplementary Information

Temporal changes in global soil respiration since 1987

Jiesi Lei[1#], Xue Guo[1#], Yufei Zeng[1], Jizhong Zhou[2, 3, 4], Qun Gao[1, 2*], and Yunfeng Yang[1*]

[1]State Key Joint Laboratory of Environment Simulation and Pollution Control, School of Environment, Tsinghua University, Beijing 100084, China;

[2]Institute for Environmental Genomics, University of Oklahoma, Norman, Oklahoma 73019, USA;

[3]Department of Microbiology and Plant Biology, University of Oklahoma, Norman, Oklahoma 73019, USA;

[4]Earth and Environmental Sciences, Lawrence Berkeley National Laboratory, Berkeley, California 94270, USA

Supplementary Table 1. Temporal changes of R_S across different ecosystems

Ecosystem type	1987-1999			2000-2016			1987-2016		
	Slope	*P*	count	Slope	*P*	count	Slope	*P*	count
Forest	-2.869	0.712	496	5.967	**0.045**	1445	3.526	0.058	1941
Deciduous forest	-11.541	0.167	197	-4.748	0.150	583	0.126	0.951	780
Evergreen forest	0.896	0.949	222	18.078	**<0.001**	817	6.940	**0.024**	1039
Mixed forest	1.008	0.969	17	-20.133	**0.040**	45	-5.503	0.331	62
Grassland	28.020	0.337	60	-1.634	0.843	210	-13.753	**0.011**	270
Shrubland	174.090	0.149	4	-6.075	0.543	132	1.726	0.848	136
Wetland	67.190	**0.002**	46	30.470	0.091	39	18.012	**<0.001**	85
Others	-97.170	0.635	7	49.840	0.045	49	8.062	0.673	56

Slopes and *P*-values of linear regressions between R_S and the measurement year are shown in the table. The number of observations used in each linear regression is also indicated. Ecosystem *Forest* comprises three types of forests, i.e., deciduous forest, evergreen forest, and mixed forest.

Supplementary Table 2. Summary of all effects in the linear model of R_S in 1987-2016.

Effect	Degree of freedom	F	P
Latitude	1	870.373	<0.001
Ecosystem type	8	11.969	<0.001
Altitude	1	54.971	<0.001
Biome×MAP	5	11.292	<0.001
MAP	1	27.558	<0.001
Biome×MAT×MAP	5	7.750	<0.001
Measurement method	1	23.567	<0.001
Biome×ΔMAT×ΔMAP	5	6.978	<0.001
MAT	1	22.107	<0.001
Biome	5	6.444	<0.001
Year×SOC	1	18.469	<0.001
Biome×MAT	5	3.982	0.001
Biome×ΔMAT	5	3.887	0.002
Year	1	9.063	0.003
ΔMAT	1	9.005	0.003
Year×Biome	5	3.077	0.009
$Year^2$	1	5.964	0.015
Year×Latitude	1	4.925	0.027
Biome×ΔMAP	5	2.480	0.030
Year×Stage	2	2.035	0.131
Year×Measurement method	1	1.578	0.209
ΔMAT×ΔMAP	1	0.668	0.414
ΔMAP	1	0.622	0.430
Year×Altitude	1	0.540	0.463
Year×Ecosystem type	7	0.745	0.634
Stage	2	0.364	0.695
SOC	1	0.110	0.740
MAT×MAP	1	0.002	0.967

The linear model was performed by year-weighted variables as described in Methods and tested with ANOVA. Effects tested include the year of R_S measurement and its quadratic form (i.e., $Year^2$), biome (i.e., tropical, subtropical, Mediterranean, temperate, boreal, and Arctic biome), measurement method, latitude, altitude, stage (aggrading or mature ecosystem), ecosystem (e.g., forest, grassland, savanna, shrubland, and wetland), SOC stock, and climatic factors (MAT, MAP, ΔMAT, and ΔMAP). The "×" sign denotes an interaction term.

Supplementary Table 3. Summary of the effects in the linear models of R_h and R_a in 1987-2016.

Effect	Degree of freedom	R_h		R_a	
		F	*P*	F	*P*
Latitude	1	252.306	<0.001	268.156	<0.001
Year^2	1	58.909	<0.001	0.044	0.835
Biome×MAT	3	8.641	<0.001	5.099	0.002
Partitioning method	11	3.962	<0.001	4.083	<0.001
Biome	4	4.516	0.001	16.405	<0.001
MAT×MAP	1	9.677	0.002	7.382	0.007
Altitude	1	6.918	0.009	14.575	<0.001
Year×SOC	1	6.594	0.011	0.132	0.716
Measurement method	1	4.833	0.029	5.457	0.020
Year	1	4.580	0.033	2.384	0.123
Biome×ΔMAT	3	2.764	0.042	1.538	0.204
Biome×MAP	3	2.280	0.079	0.763	0.515
MAT	1	2.971	0.086	47.414	<0.001
Year×Ecosystem type	5	1.886	0.096	2.092	0.066
Year×Latitude	1	2.228	0.136	4.710	0.031
Biome×MAT×MAP	3	1.571	0.196	2.359	0.071
ΔMAT	1	1.565	0.212	0.196	0.658
Biome×ΔMAT×ΔMAP	3	1.227	0.300	4.435	0.004
ΔMAP	1	0.992	0.320	5.442	0.020
Ecosystem type	5	1.154	0.331	10.536	<0.001
Year×Biome	4	1.090	0.361	3.113	0.015
Year×Altitude	1	0.708	0.401	1.797	0.181
Year×Stage	1	0.564	0.453	1.828	0.177
Year×Measurement method	1	0.393	0.531	18.088	<0.001
Biome×ΔMAP	3	0.728	0.536	1.716	0.163
Year×Partitioning method	9	0.840	0.580	3.404	<0.001
SOC	1	0.075	0.784	9.898	0.002
MAP	1	0.005	0.944	10.633	0.001
Stage	1	0.001	0.979	4.180	0.042
ΔMAT×ΔMAP	1	<0.001	0.994	4.342	0.038

A total of 468 observations of soil R_h and 473 observations of soil R_a were analyzed with the linear model controlled by year-weighted variables, as described in Methods and tested with ANOVA. Effects tested include the year of R_h or R_a measurement and its quadratic form (i.e., year^2), partitioning methods (methods used to partition R_h and R_a from R_S), biome (i.e., tropical, subtropical, Mediterranean, temperate, and boreal biome), measurement method, latitude, altitude, stage (aggrading or mature ecosystem), ecosystem (e.g., forest, grassland, savanna, shrubland, and wetland), SOC stock, and climatic factors (MAT, MAP, ΔMAT, and ΔMAP). The "×" sign denotes an interaction term.

Supplementary Table 4. Summary of *R*s change rates in periods separated by potential breakpoints

Breakpoint	Before breakpoint			After breakpoint		
	slope	*P*	count	slope	*P*	count
1996	35.32	0.006	247	-0.49	0.821	2181
1997	34.61	0.001	306	0.05	0.983	2122
1998	30.97	<0.001	385	0.53	0.824	2043
1999	27.66	<0.001	553	2.75	0.307	1875
2000	16.70	0.004	665	0.69	0.814	1763
2001	13.03	0.008	829	0.72	0.833	1599
2002	13.99	0.001	951	3.55	0.345	1477
2003	9.94	0.010	1127	4.00	0.350	1301
2004	6.85	0.038	1323	2.72	0.599	1105
2005	3.79	0.200	1469	-4.92	0.428	959
2006	4.48	0.087	1638	-6.01	0.440	790
2007	4.47	0.061	1796	-9.35	0.341	632
2008	4.57	0.032	1981	-18.75	0.197	447
2009	5.43	0.007	2100	-15.88	0.392	328
2010	4.93	0.009	2220	-38.52	0.161	208
2011	4.22	0.019	2301	-87.34	0.029	127
2012	4.27	0.013	2372	-107.89	0.153	56
2013	3.89	0.020	2414	-23.01	0.863	14
2014	3.53	0.033	2423	-269.50	0.342	5
2015	3.34	0.042	2427	NA	NA	1

Slopes, *P*-values, and the number of observations (count) of linear regressions between R_S and the measurement year in different periods are shown in the table. Each year from 1996 to 2015 was tested as a potential breakpoint.

Supplementary Table 5. Homogeneity tests for breakpoint detection

Homogeneity tests	Breakpoint	*P*
Buishand Range Test	1999	0.002
Buishand U Test	1999	<0.001
Standard Normal Homogeneity Test	2000	<0.001

Three different homogeneity tests (Buishand Range Test, Buishand U Test, and Standard Normal Homogeneity Test) were used to identify the the breakpoint of Rs change rates. The breakpoints detected and corresponding *P*-values are shown in the table.

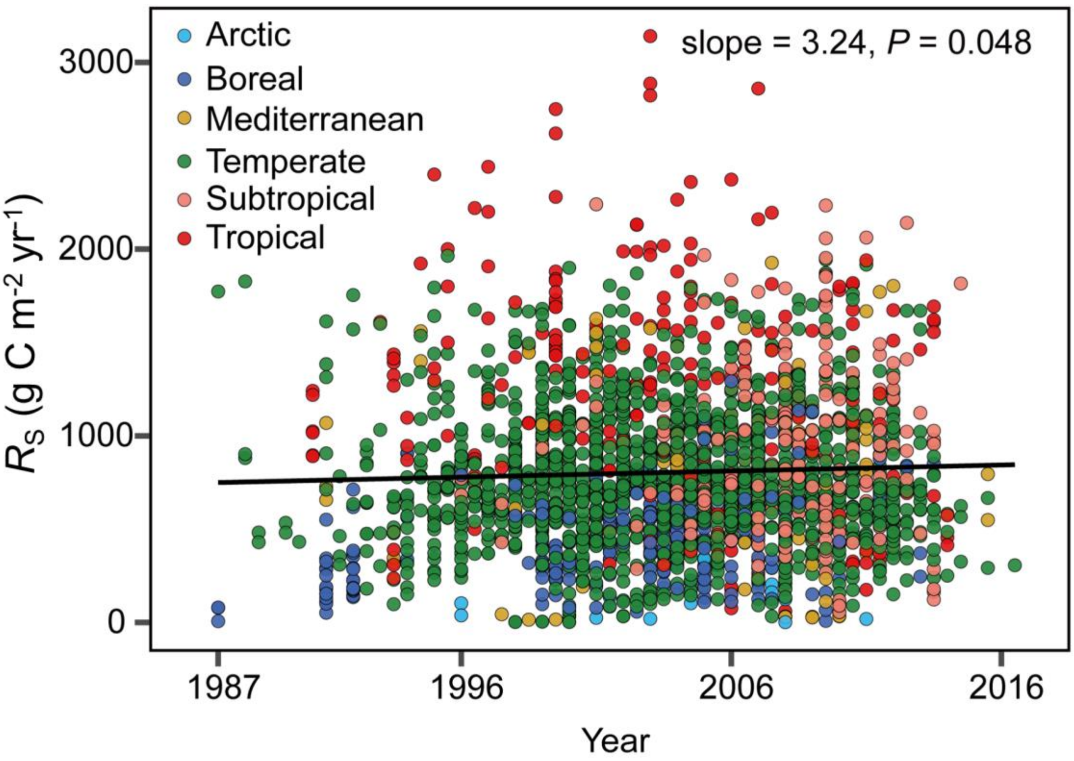

Supplementary Fig. 1 Temporal trend of R_S. The temporal distribution and trend of R_S in 1987-2016 are shown, with biomes denoted by different colors. The slope of linear regressions between R_S and year indicates the rate of R_S changes.

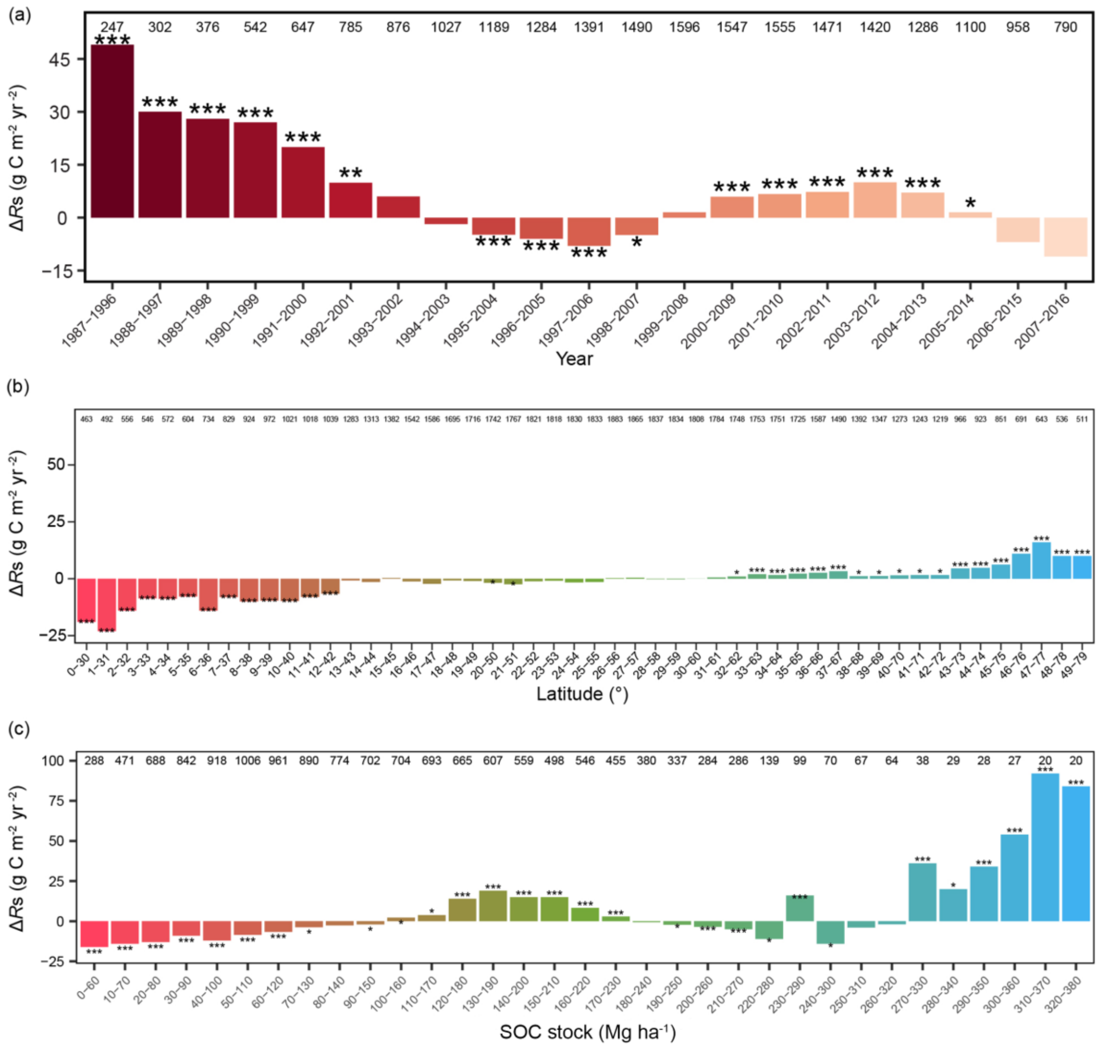

Supplementary Fig. 2 Moving window analyses using the Theil-Sen estimator. The change rate of *R*s within each window was estimated using the Theil-Sen estimator. **a**, The temporal trend of *R*s changes in each decade based on the moving subset window analysis. The bars indicate the rates of *R*s changes per decade, calculated as average annual *R*s change (Δ*R*s). **b**, Latitude dependence of *R*s changes based on the moving subset window analysis. Each window includes a subset of *R*s data within a 30° latitude interval and moves forward by 1° step. The bars represent the rates of *R*s changes in different latitudes, calculated as average annual *R*s change (Δ*R*s) in 1987-2016. **c**, Moving subset window analyses of *R*s changes in different SOC stock levels. Each window includes a subset of *R*s data whose SOC stocks are within a range of 60 Mg ha^{-1} and moves forward by 10 Mg ha^{-1} step**.** The bars represent the rates of *R*s changes in different ranges of SOC stocks calculated as average annual *R*s change (Δ*R*s) in 1987-2016. The number above each bar refers to the number of *R*s records belonging to the subset window. *** $P < 0.001$, ** $P < 0.010$, * $P < 0.050$.

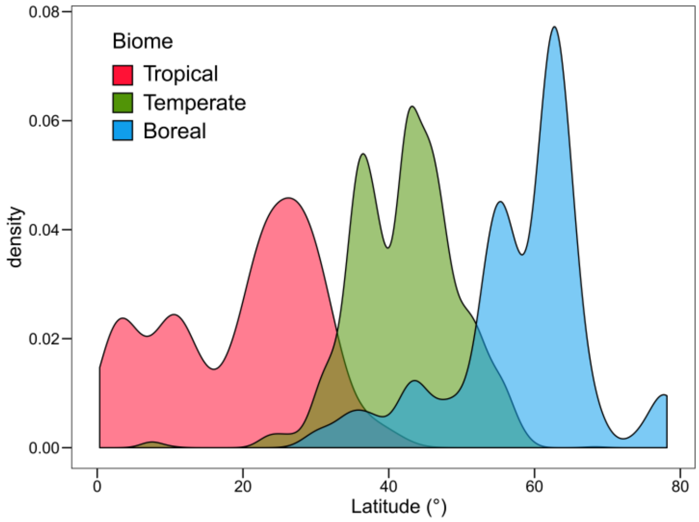

Supplementary Fig. 3 Latitude dependence of biomes. All *R*s data are classified into tropical (including data from tropics and subtropics, n = 494), temperate (including data from temperate and Mediterranean biomes, n = 1,644), and boreal (including data from boreal and Arctic biomes, n = 290). The latitudinal distributions of *R*s data in different biomes are shown.

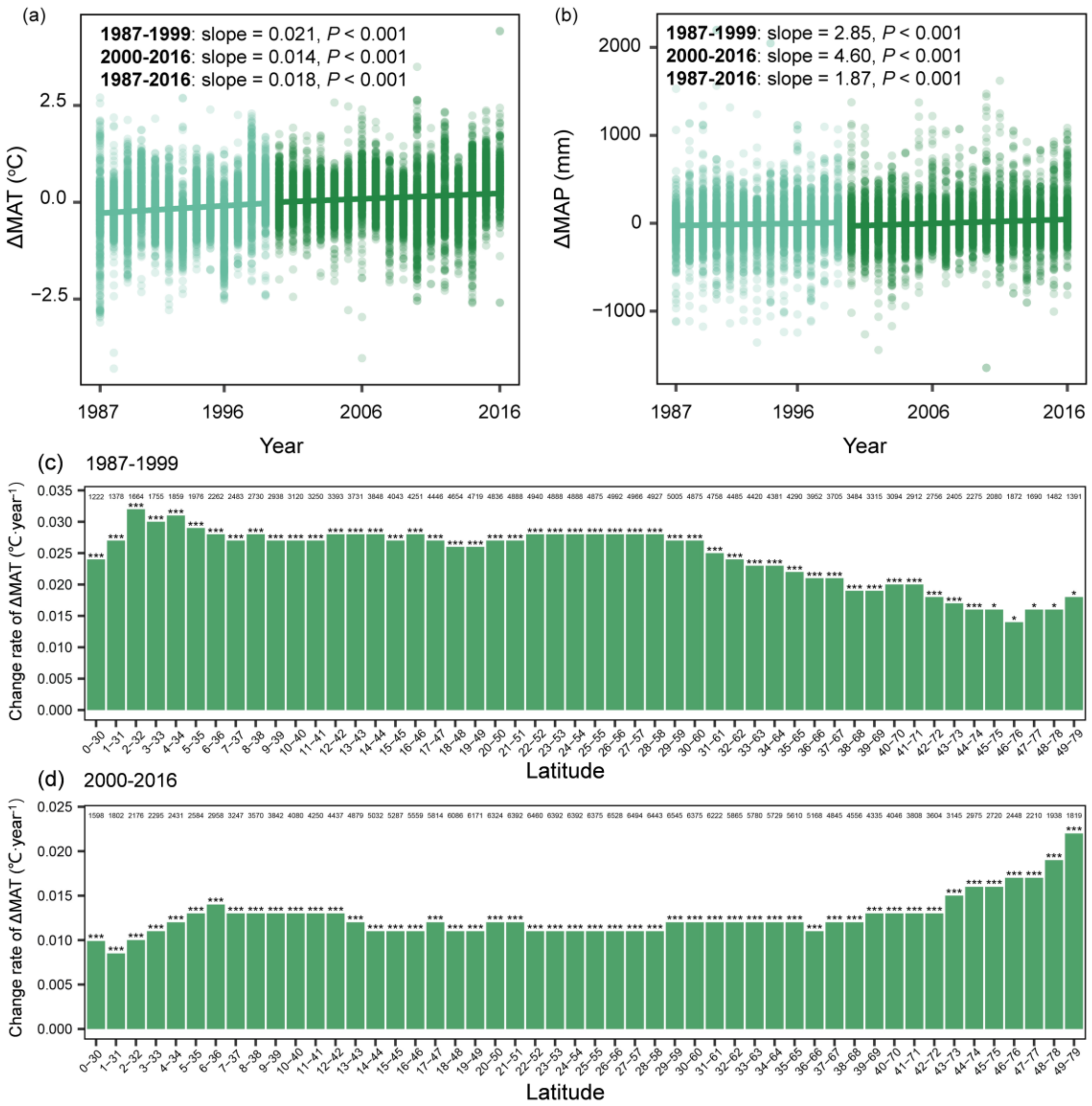

Supplementary Fig. 4 Spatiotemporal trends of temperature and precipitation anomalies. Slopes and significances of the linear regression between temperature anomalies (ΔMAT) and year **(a)**, and between precipitation anomalies (ΔMAP) and year **(b)** are shown in 1987-2016 and two time periods (1987-1999 and 2000-2016). The slopes indicate the rates of climatic anomaly changes over time. **c-d,** Latitude dependence of the rates of ΔMAT changes in 1987-1999 **(c)** and 2000-2016 **(d)** based on the moving subset window analysis. Each window includes a subset of ΔMAT data within a 30° latitude interval and moves forward by 1° step. The bars represent the rates of ΔMAT changes in different latitudes. The number above each bar refers to the number of ΔMAT records belonging to the subset window. *** $P < 0.001$, ** $P < 0.010$, * $P < 0.050$. The dot density represents data density at each point. Solid lines indicate significant trends ($P < 0.050$).

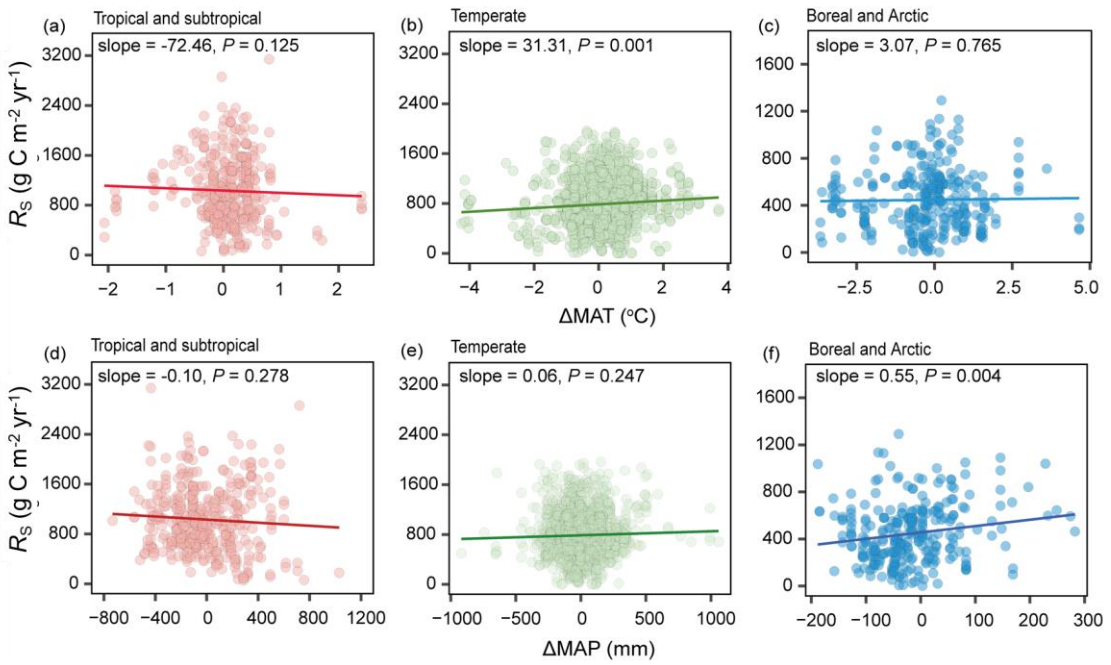

Supplementary Fig. 5 Relationship of R_S and climatic anomalies in different biomes. a-c, The relationship of R_S and temperature anomaly (ΔMAT) in tropical and subtropical biomes **(a)**, temperate and Mediterranean biomes **(b)**, and boreal and Arctic biomes **(c)**. **d-f,** The relationship of R_S and precipitation anomaly (ΔMAP) in tropical and subtropical biomes **(d)**, temperate and Mediterranean biomes **(e)**, and boreal and Arctic biomes **(f)**. Slopes and significances of linear regression are shown in the plots. The dot density represents data density at each point.

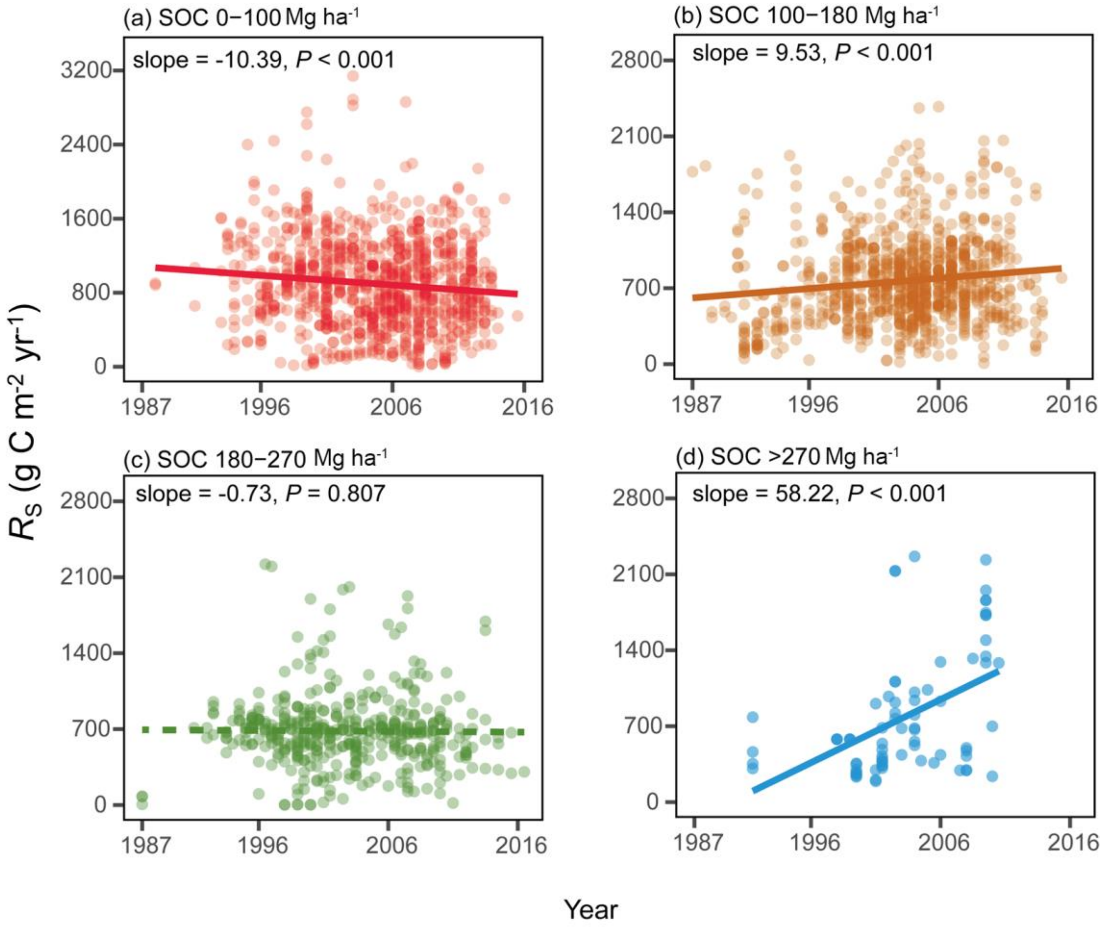

Supplementary Fig. 6 Temporal trends of $\boldsymbol{R_S}$ **in different SOC stock ranges.** The relationships of R_S and year in SOC stock ranges of 0-100 Mg ha^{-1} **(a)**, 100-180 Mg ha^{-1} **(b)**, 180-270 Mg ha^{-1} **(c)**, and above 270 Mg ha^{-1} **(d)** are fitted by the linear regression. The slopes and significances of linear regression represent the rates of R_S changes in different biomes. The dot density represents data density at each point. Solid lines indicate significant trends ($P < 0.050$), while dashed lines indicate insignificant trends ($P \geq 0.050$).
